# Meta-Analysis Using NGS Data: The *Veillonella* Species in Dental Caries

**DOI:** 10.3389/froh.2021.770917

**Published:** 2021-10-22

**Authors:** Naile Dame-Teixeira, Ana Karolina Almeida de Lima, Thuy Do, Cristine Miron Stefani

**Affiliations:** ^1^Department of Dentistry, School of Health Sciences, University of Brasilia, Brasilia, Brazil; ^2^Division of Oral Biology, School of Dentistry, University of Leeds, Leeds, United Kingdom

**Keywords:** next-generation sequencing, 16S rRNA amplicon sequencing, oral microbiology, systematic reviews, meta-analysis

## Abstract

**Objectives:** In light of recent technological advances in Next-generation sequencing (NGS) and the accumulation of large, publicly available oral microbiome datasets, the need for meta-analysing data on caries microbiome is becoming feasible and essential. A consensus on the identification of enriched organisms in cariogenic dysbiotic biofilms would be reached. For example, members of the *Veillonella* genus have been detected in caries biofilms, and may have an underestimated contribution to the dysbiotic process. Hence, we aimed to determine the abundance of *Veillonella* species in dental caries in studies using NGS data.

**Materials and Methods:** Analysis was performed according to the Preferred Reporting Items for Systematic Review and Meta-Analysis (registered at PROSPERO: CRD42020204150). Studies investigating microbial composition in saliva, dental biofilm, or carious dentin were included. Six databases and grey literature were searched. Two independent reviewers selected the papers and assessed the methodological quality.

**Results:** Searches retrieved 1,323 titles, from which 38 studies were included in a qualitative synthesis, comprising a total of 1,374 caries and 745 caries-free individuals. Most studies analysed 16S rRNA amplicons, and only 5 studies used shotgun metagenomics and metatranscriptomics. A geographical bias was observed. The methodological quality was downrated in 81.5% of the studies due to the lack of criteria for defining cases and standard criteria used for measurement of the condition in a reliable way. Six studies on early childhood caries (ECC) were meta-analysed, confirming a significant enrichment of *Veillonella* spp. in caries-associated biofilms (but not saliva) when compared to caries-free controls [mean difference: 2.22 (0.54–3.90); *p* = 0.01].

**Conclusions:** *Veillonella spp*. is more abundant in individuals suffering with ECC when compared to caries-free controls (very low evidence certainty), and should be considered for further studies to observe their metabolism in dental caries. There is an urgent need for a consensus in methodologies used to allow for more rigorous comparison between NGS studies, particularly including clinical data and details of caries diagnosis, as they are currently scarce. Inconsistent reporting on the NGS data affected the cross-study comparison and the biological connexions of the relative abundances on caries microbiome.

## Introduction

Next-generation sequencing (NGS) approaches have been key in revolutionising microbiology, and dental caries microbial communities have been widely explored with these approaches. However, challenges remain when carrying out comparative data analyses due to the differences in methodologies and protocols employed. Recently, literature on carious biofilms investigated by 16S rRNA sequencing has grown considerably, however, much of the research up to now has been descriptive in nature. The existing accounts fail to resolve the contradiction between the abundance of several microorganisms in caries and in health. Studies on either 16S rRNA amplicon or shotgun sequencing allow the investigation of hundreds of taxa in large datasets, but a lack of biological connexions can be observed between those taxa and the disease development. Specific aspects of how some species collaborate to promote caries progression has been unmanageable by describing the microbiota in high abundance enriched in disease-associated biofilms. This is information that varies widely across studies. The enrichment of some species in a dysbiotic biofilm may reflect their unique capacity to exploit their niche, particularly in being favoured by the increase in specific nutrient availability. However, are there virulence factors that promote host damage in enriched taxa? Opportunistic pathogens may contribute to the compositional and/or functional shift towards dysbiosis. To answer that question, first, it would be imperative to identify who they are.

Meta-analyses of NGS data can enable the estimation of the global prevalence of specific members of the oral microbiome in caries for example. By gathering the existing evidence on enriched microorganisms present in caries, other than *Streptococcus mutans*, dysbiotic signatures may be detected and used as biomarkers or predictors in translational research. After understanding the pattern of microbial distribution, a deeper study of their physiology could infer on health-to-disease mechanisms and have significant clinical benefits. This could facilitate the development of novel treatments by focusing therapeutic strategies on a limited number of bacterial targets which may be able to stabilise the microbial community and revert or prevent its dysbiotic state [1].

Studies exploring the role of *Veillonella* spp. in oral biofilms are surprisingly infrequent despite their ubiquity and high abundance, and this has been credited to its challenging genetic manipulation [2]. Species from this genus are not among the most studied in dental caries, although their metabolic accountability in the transition of a homeostatic to dysbiotic oral microbial community is arguable as “middle” members of the carbon food chain, meaning that its substrates are derivates of metabolic processes of microbial partners. *Veillonella* spp. are involved in important inter-species interactions with other organisms such as streptococci through co-aggregation [3–6]. They are also capable of symbiotic relations by using the organic acids produced by several oral streptococci, including *S. mutans*, which benefits the growth of both species serving as an acid sink. Additional to the interactions in biofilms, a clue of the role of *Veillonella* in the second stage of root caries development was previously observed, with high expression of genes that code for bacterial collagenolytic proteases [7]. Exploring their abundance in biofilms in health and caries conditions could be relevant to recognise its ecological significance in the complex oral biofilms. Through this systematic review and meta-analyses, we aimed to determine the abundance of *Veillonella* species in dental caries in studies using NGS methods.

## Materials and Methods

### Protocol and Registration

This systematic review was performed according to the Preferred Reporting Items for Systematic Review and Meta-Analysis (PRISMA) checklist [8]. The protocol for this study was registered at the International Prospective Register of Systematic Reviews (PROSPERO) database, under the identification number CRD42020204150.

### Eligibility Criteria

The acronym PECOs (Population; Exposition; Comparator; Outcomes and Studies) was used to design the search: Participants/population = Humans; Exposure(s) = Dental caries/root caries; Comparator(s)/control = No dental caries / no control; Outcome = Abundance of *Veillonella* at any taxonomic level (proportion, abundance, average); Studies = Observational and clinical studies applying NGS methods.

Included studies comprised the ones analysing microbial composition by NGS methods in samples (saliva, dental biofilm, or carious dentin) of individuals with dental caries, early childhood caries (ECC), or root caries, compared or not with a control group without caries. Studies eligible for this review were either observational or clinical studies.

Exclusion criteria were: (1) Animals, *in situ* or *in vitro* studies, (2) Studies written in languages not possible to be translated into an electronic translator, (3) Studies including either systemic diseases or syndromes associated with microbiota shift (Sjogren, severe hyposalivation, head and neck cancer, HIV, rheumatoid arthritis, asthma, alcoholism, etc), (4) Non-primary studies (reviews, book chapters, opinions, letters), conference abstracts, study protocols, (5) Numeric data on either prevalence or abundance of *Veillonella* spp. at any taxonomic level not specifically described, (6) Studies where the protocol for sequencing includes either a cloning step or a previous treatment, (7) A caries group was not defined, and (8) No NGS technique.

### Data Sources and Search Strategy

The search process was performed in January 2021. Appendix Table 1 shows the search strategy. “Dental caries, NGS, Oral Microbiota” were used as main search terms that were adapted for each electronic database, namely, MEDLINE *via* PubMed, LILACS, Web of Science, Scopus, Embase, Cochrane, and Livivo. Grey Literature search was also performed in Google Scholar, ProQuest, and OpenGrey. Moreover, reference lists from included studies were assessed to identify other possibly eligible studies. No language or time restrictions were applied. Duplicates were identified through *EndNoteWeb* (Clarivate Analytics, Mumbai) and then manually identified at *Rayyan QCRI*^®^ (Qatar Computer Research Institute, Qatar).

### Study Selection

The selection process was performed in two phases. First, two independent and blinded reviewers screened titles and abstracts. A previous inter-reviewer calibration was performed using 20 retrieved studies (unweighted Kappa = 0.8). This phase was carried out in a web application tool designed for systematic reviews (Rayyan *QCRI*^®^, Qatar Computing Research Institute). Any disagreement was discussed in a consensus meeting. In a second phase, the same reviewers gathered all the included studies by independently reading full articles. All selected studies had the full-text available online. Once a study was selected in the second phase and the information regarding *Veillonella* species (at any taxonomic level) was not numerically available in any way through either the full-text or supplementary material, a protocol was performed, in which an email with reminders requesting the data was sent to authors every 3 days for 15 days. Studies with either no answers from the authors or with a negative response were then excluded. Any disagreement in the data extraction was discussed with an expert and the coordinator.

### Methodological Quality Assessment

The same reviewers independently assessed the methodological quality of individual studies using the JBI Critical Appraisal Checklist for Analytical Cross-Sectional Studies [9]. For clinical studies, the same tool was applied because only the data from the baseline was considered. Due to the design of included articles, besides all eight questions of the adopted appraisal tool are considered important, two of them were considered highly critical domains to this systematic review, including “Was the exposure measured in a valid and reliable way?” and “Were objective, standard criteria used for measurement of the condition?”. A decision of excluding the studies with “no” answers for at least one of these domains was implemented. Another two were considered critical. These included: “Were the criteria for inclusion in the sample clearly defined”? and “Were the study subjects and the setting described in detail?.” Criteria related to the outcome generated by the NGS methods were considered non-critical (criteria numbers 5–8).

Criteria adopted to this systematic review for considering a low methodological quality were as follows: two “no” or one “no” and one “unclear” or two “unclear” in critical domains, or two “unclear” and one or more “no” in non-critical domains. High methodological quality was considered when an article got a maximum one “no” answer or two “unclear” answers in non-critical domains. Papers with two “no” in highly critical domains were excluded. Decision on critical and non-critical domains and classification system was discussed with the research team before the application of the instrument, as described at JBI Reviewer's Manual [9].

### Data Analysis

DerSimonian and Laird Random-Effects Meta-analysis was performed by pooling the mean values of the main outcome (relative abundance) for caries and caries-free groups and calculating the mean difference (MDs) with 95% confidence intervals. Statistical heterogeneity was estimated by the Chi-square test (*p* < 0.05) and I-squared scores (I^2^). Review Manager (RevMan; Computer program; Version 5.4) was used to conduct a meta-analysis on the relative abundance (RA) of *Veillonella* spp. in ECC vs. caries-free (subgroups of saliva, biofilm and carious dentin).

### Certainty of the Evidence

The certainty of the evidence was evaluated by the Grading of Recommendations Assessment, Development and Evaluation (GRADE) approach, performed on GRADEpro GDT [GRADEpro Guideline Development Tool (Software). McMaster University, 2015, developed by Evidence Prime, Inc., available from gradepro.org].

## Results

### Studies Selection and Methodological Quality Assessment

**Figure 1:**
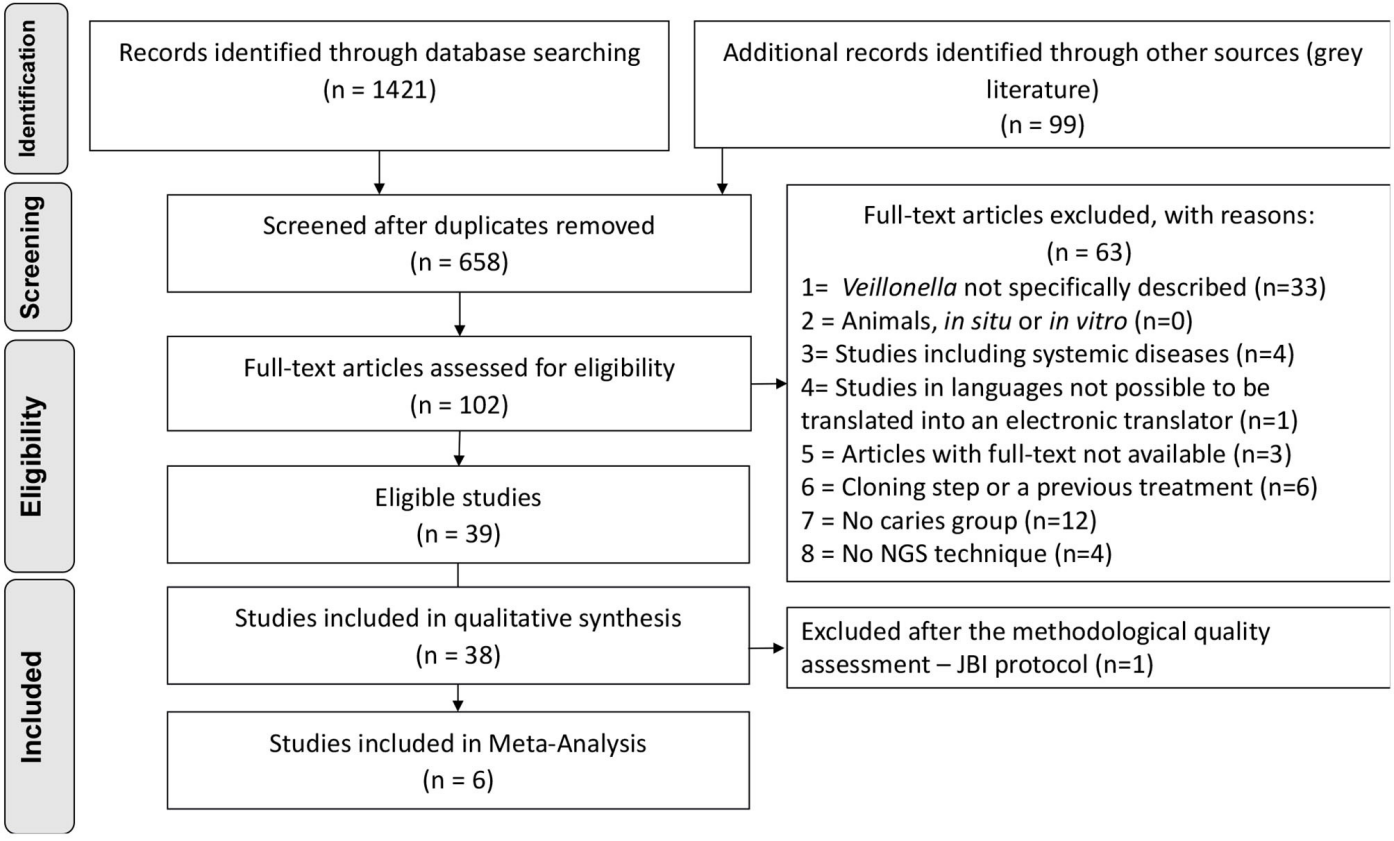
Flowchart of inclusion process, adapted from PRISMA.

Searches retrieved 1,421 titles through all databases, and 99 titles through grey literature. After duplicates removal, 658 titles remained for screening, and 102 studies continued for full-text reading. The protocol of data request was applied to 37 studies which did not specifically describe numeric data on *Veillonella* abundance at any taxonomic level. Authors from 13 studies kindly shared their data (the non-respondents were then excluded). After the complete selection process, 39 studies were eligible. The complete selection process, including reasons for exclusions, is described in the PRISMA Flowchart (Figure 1), and the list of the excluded articles can be found as supplementary data (Appendix Table 2).

The quality assessment was applied resulting in 81.6% of the articles with either moderate or low quality (*n* = 23 studies with low quality) due to the lack of description for defining cases of caries along with standard criteria used for measurement of the caries condition in a reliable way that were considered critical domains in the JBI instrument. The evaluation of caries measurement reliability was based on the description of the number of examiners, their training, and an intra or inter-examiner calibration. One article was then excluded due to “no” answers in one of the highly critical domains [10], reaching the final number of 38 studies included in qualitative synthesis (Figure 1). The overall appraisal for each included study is represented in Table 1, and detailed quality assessment as supplementary data (Appendix Table 3).

**Table 1:** Methodological characteristics of the included studies (*n* = 38).

**Author, year**	**country**	**Ref**.	**Age caries**	**Age control**	**Detection index/case definition**	**Characteristics of caries**	**Type of sample**	**Sequencing (16S rRNA region; metagenomics)**	**Methodological quality assessment**
**Caries in children**									
Agnello et al. (2017)	Canada	[11]	42.8 ± 12.2 mo.	37.4 ± 10.3 mo.	DMFT	S-ECC	1	V3–V4, Illumina	+
Al-Hebshi et al. (2019)	USA	[12]	6–10 yo.	6–10 yo.	ICCMS + X-ray	ECC; early caries (white spots) and advanced caries (at least one cavitated lesion)	1	Metagenomics, Ion S5XL	+
Chen et al. (2020)	China	[13]	6–8 yo.	6–8 yo.	WHO | DMFS	Caries in children	1; 2	V1–V3, 454 pyrosequencing	++
Dashper et al. (2019)	Australia	[14]	48.6 mo.	48.6 mo.	ICDAS II	ECC	2	V4, Ion Torrent	+
de Jesus et al. (2020)	Canada	[15]	<72 mo	<72 mo	dmft	S-ECC	1	V4, Illumina MiSeq	++
Grier et al. (2020)	USA	[16]	1–3 yo.	1–3 yo.	ICDAS	ECC	2	V1–V3, Illumina MiSeq	+
Hurley et al. (2019)	Ireland	[17]	<60 mo.	<60 mo.	WHO, ICDAS II	S-ECC	2; 3	V4–V5, Illumina MiSeq	+
Meng et al. (2015)	China	[18]	3–6 yo.	3–6 yo.	ICDAS	ECC	1	V3–V5, 454 pyrosequencing	++
Nomura et al. (2020)	Japan	[19]	9–13 yo.	9–13 yo.	WHO DMF (individuals with missing and filling teeth excluded)	Untreated caries	1	V3–V4	+
Ortiz et al. (2019)	USA	[20]	2–12 yo.	2–12 yo.	DMFT; confirmatory x-ray	Active caries in children	2	V3–V4, Illumina	+
Ribeiro et al. (2017)	Brazil	[21]	12 yo.	12 yo.	DMFT	Active white spot lesion	1	V1–V2, Illumina MiSeq	+
Richards et al. (2017)	USA	[22]	2–7 yo.	2–7 yo.	ICDAS II	Enamel and dentin caries	1	V3–V4, Illumina MiSeq	++
Tian (2015)	China	[23]	2–4 yo.	NA	DFT	ECC	1	V3–V4, Illumina MiSeq	+
Xu et al. (2018)	China	[24]	3 yo.	3 yo.	WHO DMFT	ECC	1	V3–V4, Illumina MiSeq	+++
Xu et al. (2018)	China	[25]	6–8 yo.	6–8 yo.	WHO and DMFS	Caries in young children	1; 2	V1–V3, 454 pyrosequencing	+
Xiao (2018)	USA	[26]	4.0 ± 0.9 yo.	3.8 ± 1.6 yo.	AAPD and DMFT /DMFS	S-ECC	1; 2	Illumina	+++
Xu et al. (2014)	China	[27]	<30 mo.	<30 mo.	WHO dt (no missing or filled teeth); includes white-spot	ECC	1	V1–V3, 454 pyrosequencing	++
Xu et al. (2018)	China	[28]	47.6 ± 1.2 mo.	47.4 ± 2.9 mo.	ICDAS	ECC	2	V3–V4, Illumina MiSeq	++
Zheng et al. (2018)	China	[29]	56.8 ± 8.5 mo.	57.38 ± 8.53 mo.	Caries: two or more cavitated teeth	ECC	1	V3–V4, Illumina MiSeq	+
**Caries in adolescents/adults/elderly**									
Alcaraz et al. (2012)	Spain	[30]	ND	ND	Cavitated lesions	Cavitated lesions	1	Metagenomics, 454 pyrosequencing	+
Belda-Ferre et al. (2012)	Spain	[31]	ND	ND	Group 1 = 1 to 4 active cavitated lesions (*N* = 2); Group 2 = 8 to 15 active cavitated lesions (*N* = 2)	Cavitated lesions	1	Metagenomics, 454 pyrosequencing	+
Belstram et al. (2017)	Denmark	[32]	48 (range 22–76)	NA	Moller and Poulsen Index/DMFT	High levels of coronal caries	2	V3–V4, Illumina	+++
Corralo (2018)	Brazil	[33]	ND	ND	According to Kidd and Fejerskov, 2004; at least one caries lesion either active or inactive.	Non cavitated lesions	1	Metatranscriptomics, Illumina HiSeq3000	+++
Dame-Teixeira et al. (2020)	Brazil	[34]	ND	ND	Nyvad criteria	Dentinal caries from one cervical, one occlusal, and one approximal lesion	1; 3	V3–V4, ION PGM	+
Do et al. (2015)	England	[35]	ND	ND	ND	Large occlusal soft, active carious lesions	2; 5	Metatranscriptomics, Illumina GA	+
Eriksson et al. (2017)	Sweden	[36]	17 yo.	17 yo.	DFS	ND	1; 2	V3–V4, Illumina Miseq/PacBio RS II SMRT	+
Foxman et al. (2016)	USA	[37]	1–36 yo.	NA	DMFT and active caries	ND	2; 4	V6, Illumina HiSeq	+
Jagathrakshakan et al. (2015)	India	[38]	7–52 yo.	16–52 yo.	DMFT	ND	2	V6, Ion PGM	+
Jiang et al. (2019)	China	[39]	60+ yo.	60+ yo.	DMFT	Caries in elderly	1	V3–V4, Illumina MiSeq	++
Mitwalli et al. (2019)	USA	[40]	47.3 yo.	NA	ICDAS | Root caries activity	Root caries	1	V3–V4, Illumina MiSeq	+++
Qudeimat et al. (2021)	Kuwait	[41]	64	6–9 yo.	DMFT | ICDAS	Caries in children	1	V3–V4, Illumina MiSeq	+++
Roças et al. (2016)	Brazil	[42]	16–60 yo.	NA	Radiographic analysis	Deep oclusal caries with pulp exposure	3	V4„ Illumina MiSeq	+
Schulze-Schweifing (2012)	UK	[43]	ND	NA	Carious lesion—middle or inner third of dentin	Caries in dentin	3	V1–V3, 454 pyrosequencing	+
Simon-Soro et al. (2013)	Spain	[44]	ND	NA	Nyvad 2003	Active enamel caries; cavitated dentinal caries; hidden dentinal caries	1	V1–V3, 454 pyrosequencing	+
Simon-Soro et al. (2014)	Spain	[45]	ND	NA	Enamel: chalky white, opaque, and rough; Unexposed dentin cavities were assessed radiographically	Active enamel caries; cavitated dentinal caries; hidden dentinal caries	1	V1–V4, 454 pyrosequencing	+
Xiao et al. (2016)	China	[46]	20–50 yo.	20–50 yo.	DMFT	Degrees of coronal caries extension	1	V1–V3, 454 pyrosequencing	+++
Yun et al. (2019)	China	[47]	25–80 yo.	25–80 yo.	DMFT; no filled teeth	Caries in adults and elderly	2	V3–V4, Illumina MiSeq	+
Wolff et al. (2019)	Germany	[48]	20.3–68.5 yo.	22–52 yo.	DMFT	Cavitated lesions	1	V4, Illumina MiSeq	+

*NA, Not applicable; ND, Not described; mo. , Months; yo. , years-old; S-ECC, severe early childhood caries; RA, Relative Abundance; Type of sample, (1) Supragingival biofilms; (2) Saliva; (3) Carious dentin; (4) Swab; (5) Extracted teeth; Methodological quality assessment: low quality = +; moderate quality = ++; high quality = +++*.

### Qualitative and Quantitative Synthesis

**Figure 2:**
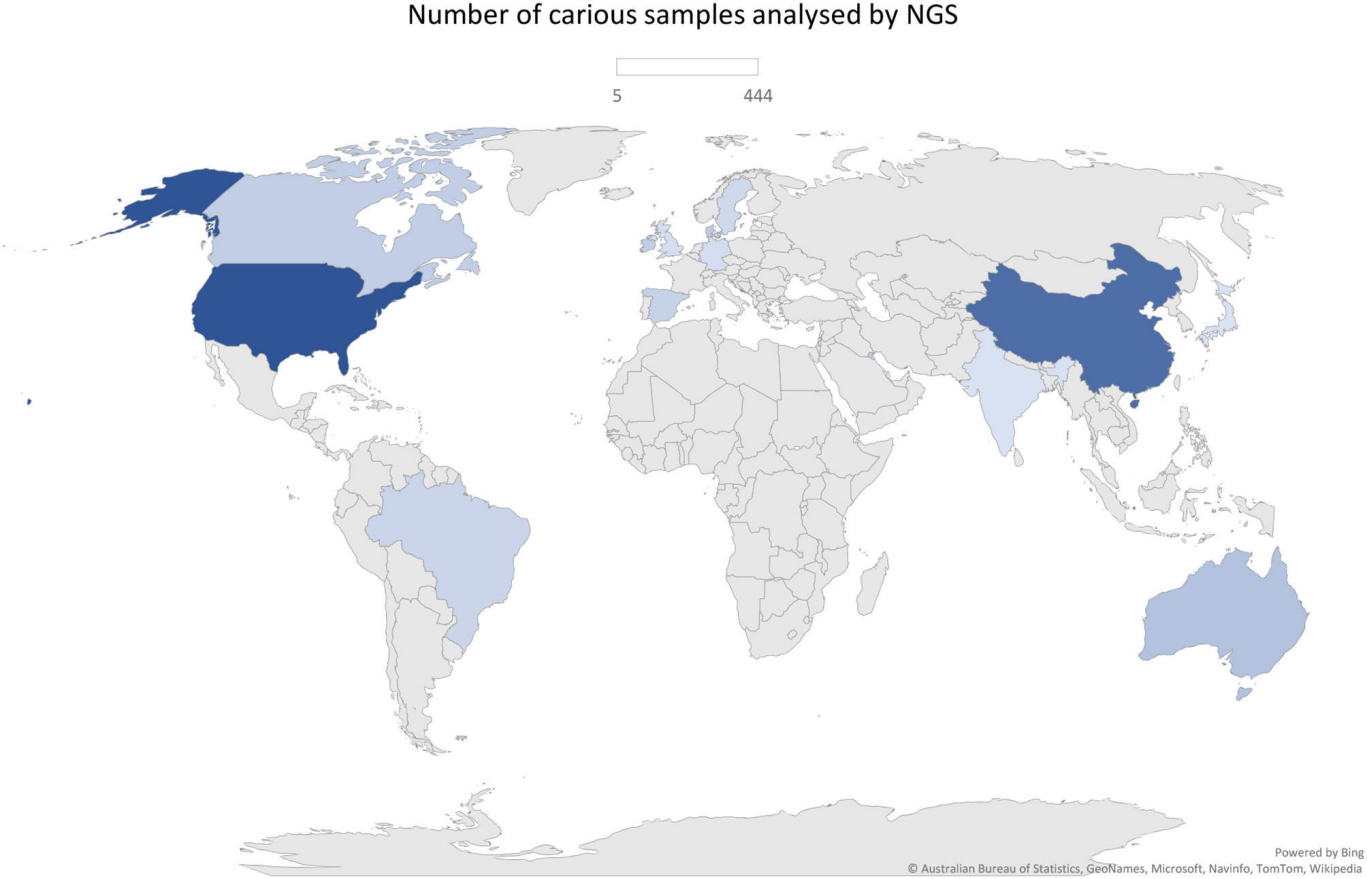
Geographical distribution of the number of samples analysed by NGS approach (*n* = 1,385).

The total of 38 studies comprised a total of 1,374 caries (*n* = 1,385 samples) and 745 caries-free individuals. Studies from several countries were included as follows: China, United States, Brazil, England, Spain, Canada, Japan, Australia, Denmark, Germany, India, Ireland, Kuwait, and Sweden. Those were published between 2014 and 2021 and are all available in the English language. Geographical patterns of *Veillonella* distribution in carious biofilms could not be found, although a significantly large number of samples was analysed in only a few countries, which can give rise to a geographical or regional bias. No NGS data on caries from African countries were found, for example (Figure 2). Most studies sampled carious-associated supragingival biofilms (24 studies). Saliva and carious dentin were also frequently sampled, and sometimes combined with analysis of biofilms samples from the same individuals (Table 1). The sample size ranged from 2 to 153 patients per group (median *n* = 20 for caries and *n* = 14 for caries-free groups).

A serious weakness across most studies was the lack of description of caries detection criteria and case definition. Only few studies clearly described the caries detection criteria and the conditions of the clinical examination (artificial light, position of the individuals, tooth cleaning and drying conditions, instruments used, etc.). When described, generally, the decayed, missing, and filled teeth (DMFT) criteria from the World Health Organisation (WHO) and the International Caries Detection and Assessment System (ICDAS) were applied (Table 1). Only four studies implemented X-ray as a confirmatory method for caries diagnosis [12, 20, 42, 45] and numerous studies used the DMFT index considering only the presence of cavities to define cases.

Although several differences were observed in both caries diagnosis and sampling, a pattern was observed when molecular methods were used to measure the microbial outcome. The efficacy of DNA/RNA extraction methods could lead to differences in microbial abundances between studies adding some bias to this comparison, however, all but three studies used commercial kits. Illumina sequencers were employed in 22 studies (predominantly MiSeq), and 16S rRNA amplicons of V1-V3 and V3-V4 regions were typically sequenced (Table 1). Only 3 [12, 30, 31] and 2 [33, 35] studies used shotgun metagenomics and metatranscriptomics to analyse the RA of microorganisms and those that are metabolically active, respectively. As seen in Table 2, transcriptomic studies detected higher abundance of *Veillonella* than the ones sequencing DNA including 16S rRNA amplicons. Regarding the sampling site, higher abundance of *Veillonella* in supragingival biofilms and carious dentin compared to saliva may indicate its site-specialisation.

**Table 2:** Relative abundance of *Veillonella* at different taxonomy levels in caries and caries-free groups.

**Author, year**	**N caries**	**N caries-free**	**Relative abundance—caries**	**Relative abundance—caries-free**
***Veillonella spp***.				
**DNA/16S rRNA**				
Agnello (2017)	30	20	4.1% (0.39–19.8)	2.4% (0.18–10.1)
Alcaraz (2012)	4	2	10.29 ± 3.81%	6.60 ± 0.98%
Al-Hebshi (2019)	20	10	Early caries: 6.44 ± 2.6%	3.38 ± 0.017%
			Advanced caries: 5.5 ± 1.35%	
Belstram (2017)	79	NA	0.16%	NA
Dame-Teixeira (2020)	3	2	Cervical lesion: 1.43%	1.19 ± 1.42
			Occlusal lesion: 2.29%	
			Approximal lesion: 18.32%	
de Jesus (2020)	40	40	0.1146 ± 0.09013	0.04621 ± 0.03047
Foxman (2016)	173	NA	0.11 ± 0.05	0.07079 ± 0.04082
Hurley (2019)	68	70	Saliva: 1.908 (1.059–3.054)	Saliva: 0.8679 (0.5101–1.652)
			Carious dentin: 2.282 (1.504–3.127)	
Jiang (2019)	24	22	7.51 ± 4.42%	8.12 ± 7.11%
Mitwalli (2019)	20	NA	5.33%	NA
Ortiz (2019)	64	21	*Veillonella* genus probe 2: 0.90%	*Veillonella* genus probe 2: 0.76%
			*Veillonella* genus probe 1: 0.08%	*Veillonella* genus probe 1: 0.08%
			*Veillonella* sp HOT 780: 0.51%	*Veillonella* sp HOT 780: 0.37%
Qudeimat (2021)	64	64	2.60%	2.20%
Ribeiro (2017)	13	13	2.2 ± 1.3%	ND
Roças (2016)	10	NA	1.30%	NA
Schulze-Schweifing (2012)	5	NA	0.04 ± 0.07	NA
Simon-Soro (2013)	22	NA	White spot: 26.27 ± 22.86	NA
			Cavitated 30.73 ± 17.57%	
			Hidden 15.09 ± 16.64%	
			All cavities: 24.77 ± 20.56%	
Xiao (2016)	131	29	1.70%	ND
Xiao (2018)	21	18	27%	22%
Xu and Chen (2018)	12	11	12.2 ± 6.4%	18.7 ± 15.6%
Xu and Jia (2018)	30	10	Saliva: 0.021 ± 0.0003%	Saliva: 0.029 ± 0.00%
			Biofilm: 0.044 ± 0.0004%	Biofilm: 0.03 ± 0.0007%
Yun (2019)	40	10	0.03% (average from 4 caries groups)	0%
Xu and Hao (2014)	10	9	11.3 ± 4.91%	8.53 ± 9.33%
Tian (2015)	24	NA	3.94 ± 5.09%	NA
Wolff (2019)	19	37	8.36 ± 5.34%	5.54 ± 4.45%
Xu and Tian (2018)	19	10	0.98 ± 2.12%	0.33 ± 0.62%
Total	**965**	**408**		
**RNA**				
Corralo (2018)	10	6	Caries-active: active site: 6.59	2.43%
			Inactive-site: 6.40%	
			Sound-site: 4.47%	
Belda-Ferre (2012)	4	2	11.393 ± 2.619%	4.315 ± 0.742%
Simon-Soro (2014)	Non-cavited enamel lesions: 15 | dentin caries lesions: 12	NA	White spot: 21.17 ± 14.86 Cavitated 10.86 ± 4.70 Hidden 13.19 ± 9.88	NA
Total	**29**	**8**		
Total *Veillonella spp*.	**994**	**416**		
**Veillonella parvula**				
DNA/16S rRNA				
Eriksson (2017)	37	26	1.13%	1.32%
Ribeiro (2017)	13	13	1.6 ± 1.4%	ND
Roças (2016)	10	NA	1.30%	NA
Total	**60**	**39**		
**RNA**				
Do (2015)	**11**	**11**	16.62 ± 11.17	4.76 ± 7.21
Total *V. parvula*	**71**	**50**		
*Veillonella dispar*				
**DNA/16S rRNA**				
Agnello (2017)	30	20	3.0% (0.33–19.0%)	2.2% (0.15–9.4%)
Eriksson (2017)	37	26	2.44%	2.63%
Meng (2015)	20	10	13.2% of the total	ND
Nomura (2020)	5	7	3.39 ± 2.62%	0.4027 ± 0.6048%
Ortiz (2019)	64	21	0.36%	0.16%
Zheng (2018)	16	15	0.11%	0.01%
Mitwalli (2019)	20	NA	5.40 ± 0.045%	NA
Total	**172**	**89**		
**RNA**				
Do (2015)	**11**	**11**	2.18 ± 1.13%	7.08 ± 5.07%
Total *V. dispar*	**183**	**100**		
***Veillonella atypica***				
**DNA/16S rRNA**				
Eriksson (2017)	37	26	0.06%	0.25%
Mitwalli (2019)	20	NA	0.30 ± 0.01%	NA
Ortiz (2019)	64	21	0.38%	0.31%
Qudeimat (2021)	64	64	2.60%	2.20%
Total	185	111		
**RNA**				
Do (2015)	11	11	0.91 ± 0.43%	4.09 ± 3.47%
Total *V. atypica*	196	122		

*Only data clearly described in the text in proportions were included in averages calculation. Lesions representing different patients from the same study were both included in the average. Bold values represents the total*.

Although some studies showed data on other species, the most commonly described were *Veillonella parvula, Veillonella dispar*, and *Veillonella atypica*. Belstram et al. [32] showed data regarding *Veillonella rogosae* (RA = 0.6%) and *Veillonella denticariosi* (RA = 0.11%) in saliva of individuals with high levels of coronal caries. Ortiz et al. [20] analysed saliva of children with active caries lesions and a control group without caries and showed an RA of *V. rogosae* of 0.21% in caries and 0.54% in caries-free. When data on species were available, *V. parvula* and *V. dispar* had higher abundance than *V. atypica* and other species. By analysing those species altogether, Dashper et al. [14] showed a difference of RA = 0.15 between caries and caries-free groups. Furthermore, it seems that *V. dispar* and *V. atypica* RA drop sharply in caries environment, while *V. parvula* was enriched in dental caries in dentinal caries lesions analysed by RNA-seq (16.62%). It is possible to qualitatively compare these data with the study by Roças et al. [42], which also evaluated carious dentin and presented a much lower proportion of *V. parvula* (1.30%) in carious dentin.

**Figure 3:**
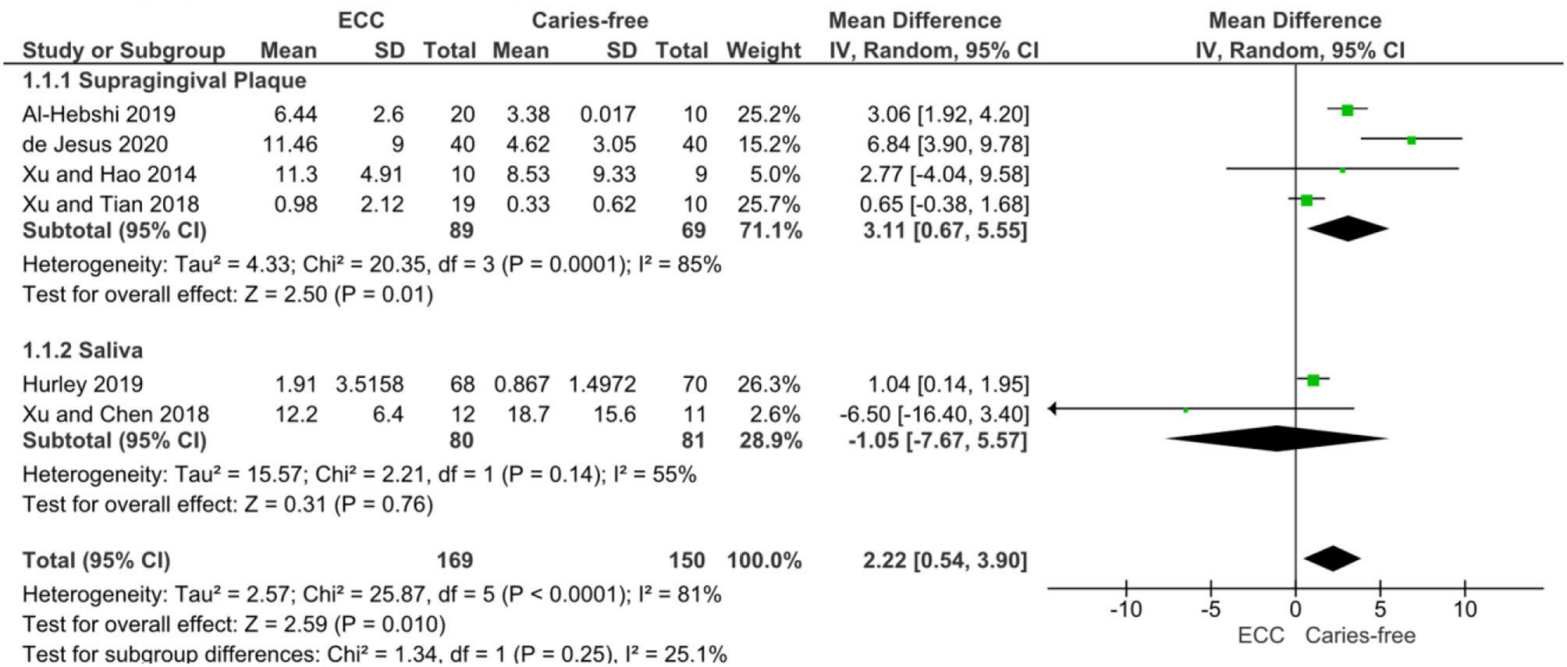
DerSimonian and Laird Random-Effects Meta-analysis of *Veillonella* spp. abundance in Early Childhood Caries (ECC) individuals compared to caries-free individuals. A subgroup analysis was performed for studies on supragingival plaque (1.1.1) and saliva (1.1.2), as well as the pooled results.

The numerical range of the RA is in the magnitude of 10^−2^ to 10^2^ (Table 2), and it might be related either to methodological or differences in data analysis and reporting. However, the meta-analysis of six studies on ECC confirmed a significant enrichment of *Veillonella* spp. in children with caries when compared to caries-free controls [mean RA difference: 2.22 (0.54–3.90); *p* = 0.01] with considerable statistical heterogeneity (I^2^ 81%) despite one study which found conflicting results by analysing the salivary microbiome [28]. In the analysis of subgroups, the difference was clearly defined by the studies on supragingival biofilms, and the salivary enrichment of *Veillonella* in caries could not be observed (Figure 3).

Certainty of evidence evaluated through GRADE approach was rated as “very low” for *Veillonella* RA in ECC individuals compared with caries-free individuals. Considering that observational studies initiate the GRADE assessment as “low,” the certainty was further downgraded by one additional point due to low-quality of two included studies [12, 17] after sensitivity analysis (since the removal of these two low-quality studies from meta-analysis resulted in summary effect size modification—MD 2.07 CI 95% −2.44 to 6.58, *p* > 0.05). The other GRADE approach domains were not downgraded (inconsistency, indirectness, imprecision, and publication bias), nor upgraded (great magnitude, dose-response, residual confounders). Appendix Table 4 shows the summary of GRADE of finding table with detailed decisions.

## Discussion

In light of recent technological advances in NGS and the accumulation of large and publicly available oral microbiome datasets, the need for meta-analysing data on caries microbiome is becoming essential. Developing a versatile analysis pipeline for the raw sequencing data of a wide range of studies would be particularly complex due to the heterogeneity of the datasets (differences in nucleic acid isolation, library preparation, sequencing platforms used, etc.) and their associated information on clinical data. Systematic reviews can, however, be a worthy strategy to achieve a cross-study comparison of the microbiome using NGS data, as we validated here. Although a meta-analysis approach is still subject to heterogeneity, it can nevertheless be feasible. As far as we know, this is the first meta-analysis of the NGS data on dental caries. After a comprehensive literature search, 39 studies presenting *Veillonella* data from caries samples were eligible, of which 38 and 6 were qualitatively and quantitatively analysed, respectively. We demonstrated a significant enrichment of *Veillonella* spp. in ECC samples when compared to samples from caries-free samples, yet with very low certainty. However, the most striking result here is lack of clarity in defining cases and controlling confounding factors, and the need for changes in the reports on the clinical data from the donors.

Here we showed differences in *Veillonella* proportion in different lesions and type of sampling, being the supragingival biofilms more prone to demonstrate differences in RA in health-to-disease. This higher abundance of *Veillonella* in supragingival biofilms and carious dentin compared to saliva may indicate its site-specialisation. They are evidently likely to benefit from the ecological conditions of low pH environment commonly observed in carious lesions. However, *Veillonella* spp. enrichment can also suggest their non-negligible role in caries dysbiosis. A relevant research question could perhaps consider *Veillonella* spp. to have virulence potential in caries, particularly in ECC. *Veillonella* spp. are considered commensal bacteria present in the oral cavity and the gastrointestinal tract of humans, due to being part of the core microbiome [49, 50]. They are known to be involved in cross-feeding and co-aggregation with acidogenic species in other environments [51], using their metabolic intermediates, which suggest that they act as an acid sink. However, in an *in vitro* study, it was shown that *Veillonella*, when associated with *S. mutans*, did not increase the biofilm pH as expected, as well as promote the growth and extracellular polysaccharide synthesis [49]. We believe that the identification of these virulence factors and a more detailed molecular and functional characterisation of *Veillonella* in caries-associated biofilms, in combination with a more thorough characterisation of other microorganisms, could be the key for the development of strategies to modulate the microbiota by using pre or probiotics. A more recent molecular characterisation of oral *Veillonella* revealed that the genomes of oral *Veillonella* species were remarkably diverse, and that these *Veillonella* have conserved pathways that utilise carbohydrates other than lactate as an energy source [50], which might be an interesting finding of their potential pathogenic traits as oral species. Furthermore, RNA sequencing seems to show higher abundances of *Veillonella* than DNA sequencing. Although the result should be interpreted with caution as no statistical analysis was achievable. In addition, it could also suggest some inactive or dead cells in the DNA studies. Benítez-Páez showed important differences in the RA of bacterial genera from metagenomic and metatranscriptomic data by analysing dental biofilms, and *Veillonella* was one of the most commonly found in the total DNA-based metagenome, however, with a slightly lower value in RNA-based metatranscriptomics [52].

Important limitations on the heterogeneity of the primary studies should be considered. There is a concerning pattern of lack of thorough definition and description of caries cases, even for the same condition, occurring in ECC which is defined differently throughout studies. Most studies used the standard criterion of the World Health Organisation (the classic DMFT), or solely the presence of cavities to define cases. However, this can add an important bias due to the overlooking of non-cavitated caries and caries activity. Furthermore, several studies did not describe whether the “caries-free” group had past caries experience, such as the presence of surfaces with inactive lesions, fillings, or missing teeth, making unclear if this could impact the microbial composition in the microbiome after caries management and arrestment. We hypothesise the presence of a “scarf of dysbiosis” in biofilms from individuals with past caries experience by observing findings from Corralo where microbial profiles of inactive caries-associated biofilms were unlike those of caries-free biofilms [33]. Different detection criteria should be used to assess dental caries in a more accurate way, such as the Nyvad Criterion [53] and the International Caries Detection and Assessment System (ICDAS) [54]. The criterion to determine cases plays an important role in investigating caries-associated microbiota. Furthermore, most studies did not evaluate disease in the same way and did not address the calibration of dental examiners, which would have improved the certainty of the evidence in the field. New studies on caries microbiome should have plans and strategies for a better definition of caries.

Despite advances in the 16S rRNA sequencing and the shotgun whole-genome metagenomic methods, there is an urgent need to define patterns in methodological data from studies on caries microbiology to make them comparable. Although our search found a considerable number of primary studies, it was extremely complex and difficult to make a synthesis within the current available data from the literature. The microbial composition was shown in different perspectives and taxonomic levels. Other sources of variation, such as the complexity of the related clinical elements in each study, may have a great impact on the overall microbiome investigation and is often omitted in many studies. Examples include the lack of the clarity at the selection criteria and the control of confounding factors. In the present study, 20 out of 39 studies were downrated at the JBI domain: “Were confounding factors identified?”. Those confounding factors could include a large range of age, gender, patient habits (including diet, smoking, oral hygiene practises, etc.), caries extent, caries activity, salivary or biofilm pH, salivary flow, other oral conditions (gingivitis, periodontitis, tooth loss, use of prosthesis), systemic diseases, etc. We believe that further study should provide those details as they will favour more rigorous and accurate analyses of the oral microbiome, particularly when saliva is sampled. Zaura et al. already described the need for improving the clinical characterisation in future NGS studies on the oral microbiome so that the quality of the scientific literature would be improved throughout more carefully designed papers [55].

We also identified a lack of standardisation in reporting this type of study, suggesting the need of a comprehensive guideline checklist development. Reports on NGS data following guideline checklists, such as the STROBE for observational studies [56] or the CONSORT for randomised clinical trials [57] promote research reproducibility, better-quality study designs, and help the development of meta-analyses. There is a need for a specific checklist for microbiological data as we faced difficulties in obtaining accurate *Veillonella* spp. RA data from studies. Authors were requested to share their data on *Veillonella* RA and the response rate was 34%, resulting in information losses that add a selective reporting bias. Although it would be more reasonable to meta-analyse several microorganisms as caries is a polymicrobial disease, it is not logistically easy due to the differences in these reports. In order to overcome this problem, additional tables containing the average RA of all species might be uploaded as a supplementary material in further studies. Besides, not all studies that presented data on *Veillonella* clearly defined the unit used for the RA. On a positive note, the laboratory experiments and bioinformatic pipelines were adequately described in all studies.

Zhou et al. lay emphasis on the need to optimise and standardise metagenomic studies [58] as technical or methodologic heterogeneity produces systematic biases that could obscure biologically meaningful information on the compositional differences. More homogeneous studies regarding the DNA/RNA extraction methods, amplicons region sequencing, pipeline (ASV or OTUs), etc. [59] ought to lead to additional meta-analyses. Another important bias control is to develop a sample size calculation, since none of the studies retrieved here presented, thus reducing their external validity (generalisation). Low sample sizes (median of 20 individuals sampled) can be explained due to the high costs of the NGS studies. However, power calculation methods for microbiome studies are now available [60], and specific sample sizes based on the prevalence of microorganisms can also be estimated.

In order to overcome the apparent geographical bias, with data originating from selected few countries (only 14), those with perhaps greater facilities and funds for conducting NGS research, new multicentric studies, in particular, should be developed with a wider interest in the microbiomes of all human populations and ethnicities. More meta-analyses of NGS data investigating other microorganisms (or a group of) in dental caries would help gather and confirm invaluable evidence of enriched microorganisms, such as *Veillonella* spp. Understanding the pattern of their distribution and physiology could help elucidate the mechanism of the health-to-disease transition, with crucial information on colonisation, survival, and interactions, which would help define research priorities in the field of caries microbiology.

## Conclusions

*Veillonella* spp. are more abundant in individuals suffering with ECC when compared to caries-free controls (very low evidence certainty), and should be considered in further studies to better understand their metabolism and contribution to dental caries. There is an urgent need for a consensus in methodologies used to allow for more rigorous comparison, particularly including clinical data and details of caries diagnosis, which are currently scarce. Inconsistent reporting on the NGS data affected the cross-study comparison and the biological connexions of the relative abundances on caries microbiome.

## Data Availability Statement

The original contributions presented in the study are included in the article/Supplementary Material, further inquiries can be directed to the corresponding author.

## Author Contributions

ND-T contributed to conception, design, data acquisition and interpretation, drafted, and critically revised the manuscript. AL contributed to design, data acquisition and interpretation, drafted, and critically revised the manuscript. TD contributed to conception, design, and critically revised the manuscript. CS contributed to conception, design, data acquisition and interpretation, performed statistical analysis, drafted, and critically revised the manuscript. All authors gave their final approval and agree to be accountable for all aspects of the work.

## Conflict of Interest

The authors declare that the research was conducted in the absence of any commercial or financial relationships that could be construed as a potential conflict of interest.

## Publisher's Note

All claims expressed in this article are solely those of the authors and do not necessarily represent those of their affiliated organizations, or those of the publisher, the editors and the reviewers. Any product that may be evaluated in this article, or claim that may be made by its manufacturer, is not guaranteed or endorsed by the publisher.
