# Supplementary material for: Meta-Analysis Using NGS Data: The Veillonella Species in Dental Caries
Source: Front Oral Health. 2021 Oct 22;2:770917. doi: 10.3389/froh.2021.770917 (PMC8757819; doi:10.3389/froh.2021.770917)
Supplement: Supplementary file 1 [file Table_1.DOCX]

**Appendix table 1.** Search strategies according to each database, performed on 29/7/2020 and updated in 10/1/21

| Database | Search strategy | N |
| --- | --- | --- |
| Pubmed via Medline | (((((((((((("dental caries"[MeSH Terms] OR "dental caries"[All Fields]) OR "Dental Decay"[All Fields]) OR "Carious Dentin"[All Fields]) OR ((("dental caries"[MeSH Terms] OR ("dental"[All Fields] AND "caries"[All Fields])) OR "dental caries"[All Fields]) OR ("carious"[All Fields] AND "dentins"[All Fields]))) OR ((((((("teeth s"[All Fields] OR "teeths"[All Fields]) OR "tooth"[MeSH Terms]) OR "tooth"[All Fields]) OR "teeth"[All Fields]) OR "tooth s"[All Fields]) OR "tooths"[All Fields]) AND (((("carie"[All Fields] OR "dental caries"[MeSH Terms]) OR ("dental"[All Fields] AND "caries"[All Fields])) OR "dental caries"[All Fields]) OR "caries"[All Fields]))) OR "teeth caries"[All Fields]) OR "tooth caries"[All Fields]) OR "teeth decay"[All Fields]) OR "tooth decay"[All Fields]) OR "Dental Decay"[All Fields]) OR "Root Caries"[MeSH Terms]) OR "Cervical Caries"[All Fields]) AND (("sequence analysis, rna"[MeSH Terms] OR (((("sequence analysis, rna"[MeSH Terms] OR (("sequence"[All Fields] AND "analysis"[All Fields]) AND "rna"[All Fields])) OR "rna sequence analysis"[All Fields]) OR ("rna"[All Fields] AND "sequencing"[All Fields])) OR "rna sequencing"[All Fields])) OR (((((((((((("high-throughput nucleotide sequencing"[MeSH Terms] OR "high-throughput nucleotide sequencing"[All Fields]) OR "high-throughput nucleotide sequencing"[All Fields]) OR "next generation sequencing"[All Fields]) OR "Deep Sequencing"[All Fields]) OR "high throughput rna sequencing"[All Fields]) OR "high throughput rna sequencing"[All Fields]) OR "high throughput dna sequencing"[All Fields]) OR "high throughput dna sequencing"[All Fields]) OR "16S"[All Fields]) OR "16S rRNA"[All Fields]) OR "16S rDNA"[All Fields]) OR "rna, ribosomal, 16s"[MeSH Terms])) | 334  +  55 |
| Cochrane | ("dental caries" OR "Dental Decay" OR "Carious Dentin" OR "Carious Dentins" OR "teeth carie" OR "teeth caries" OR "tooth caries" OR "teeth decay" OR "tooth decay" OR "dental decay" OR "Root Caries" OR "Cervical Caries") in Title Abstract Keyword AND ("sequence analysis, rna" OR RNA-sequencing) OR ("high-throughput nucleotide sequencing" OR "high throughput nucleotide sequencing" OR "next generation sequencing" OR "Deep Sequencing" OR "High-Throughput RNA Sequencing" OR "High Throughput RNA Sequencing" OR "High-Throughput DNA Sequencing" OR "High Throughput DNA Sequencing" OR "16S" OR "16S rRNA" OR "16S rDNA" OR “RNA, Ribosomal, 16S) in Title Abstract Keyword - (Word variations have been searched) | 0  +  0 |
| Livivo | KW=(("dental caries" OR "Dental Decay" OR "Carious Dentin" OR "Carious Dentins" OR "teeth carie" OR "teeth caries" OR "tooth caries" OR "teeth decay" OR "tooth decay" OR "dental decay" OR "Root Caries" OR "Cervical Caries")) AND KW=(("sequence analysis, rna" OR RNA-sequencing) OR ("high-throughput nucleotide sequencing" OR "high-throughput nucleotide sequencing" OR "high throughput nucleotide sequencing" OR "next generation sequencing" OR "Deep Sequencing" OR "High-Throughput RNA Sequencing" OR "High Throughput RNA Sequencing" OR "High-Throughput DNA Sequencing" OR "High Throughput DNA Sequencing" OR "16S" OR "16S rRNA" OR "16S rDNA" OR “RNA, Ribosomal, 16S)) | 126  +  6 |
| Web of Science | TÓPICO: ("dental caries" OR "Dental Decay" OR "Carious Dentin" OR "Carious Dentins" OR "teeth carie" OR "teeth caries" OR "tooth caries" OR "teeth decay" OR "tooth decay" OR "dental decay" OR "Root Caries" OR "Cervical Caries") AND TÓPICO: ("sequence analysis, rna" OR "RNA-sequencing" OR "high-throughput nucleotide sequencing" OR "high throughput nucleotide sequencing" OR "next generation sequencing" OR "Deep Sequencing" OR "High-Throughput RNA Sequencing" OR "High Throughput RNA Sequencing" OR "High-Throughput DNA Sequencing" OR "High Throughput DNA Sequencing" OR "16S" OR "16S rRNA" OR "16S rDNA" OR "RNA, Ribosomal, 16S") | 247  +  39 |
| Scopus | ( TITLE-ABS-KEY ( "dental caries" OR "Dental Decay" OR "Carious Dentin" OR "Carious Dentins" OR "teeth carie" OR "teeth caries" OR "tooth caries" OR "teeth decay" OR "tooth decay" OR "dental decay" OR "Root Caries" OR "Cervical Caries" ) AND TITLE-ABS-KEY ( "sequence analysis,rna" OR "RNA-sequencing" OR "high-throughput nucleotide sequencing" OR "high throughput nucleotide sequencing" OR "next generation sequencing" OR "Deep Sequencing" OR "High-Throughput RNA Sequencing" OR "High Throughput RNA Sequencing") ) | 107  +  23 |
| Embase | ('dental caries'/exp OR 'dental caries' OR 'carious dentin' OR 'carious dentins' OR 'teeth carie' OR 'teeth caries' OR 'tooth caries' OR 'teeth decay' OR 'tooth decay' OR 'dental decay' OR 'root caries' OR 'cervical caries') AND ('high throughput sequencing'/exp OR 'rna 16s'/exp OR 'sequence analysis, rna' OR 'rna-sequencing' OR 'high-throughput nucleotide sequencing' OR 'high throughput nucleotide sequencing' OR 'next generation sequencing' OR 'deep sequencing' OR 'high-throughput rna sequencing' OR 'high throughput rna sequencing' OR 'high-throughput dna sequencing' OR 'high throughput dna sequencing' OR '16s' OR '16s rrna' OR '16s rdna' OR 'rna, ribosomal, 16s') | 397  +  73 |
| LILACS via BVS | tw:((tw:(("dental caries" OR "Dental Decay" OR "Carious Dentin" OR "Carious Dentins" OR "teeth carie" OR "teeth caries" OR "tooth caries" OR "teeth decay" OR "tooth decay" OR "dental decay" OR "Root Caries" OR "Cervical Caries" OR “cárie dentária” OR “caries dental” OR “cárie dental” OR cáries OR “cáries dentais” OR “cáries dentárias” OR “dente cariado” OR caries OR “caries dentales”) )) AND (tw:("sequence analysis, rna" OR “rna-sequencing” OR "high-throughput nucleotide sequencing" OR "high throughput nucleotide sequencing" OR "next generation sequencing" OR "Deep Sequencing" OR "High-Throughput RNA Sequencing" OR "High Throughput RNA Sequencing" OR "High-Throughput DNA Sequencing" OR "High Throughput DNA Sequencing" OR "16S" OR "16S rRNA" OR "16S rDNA" OR “rna, ribosomal, 16s” OR “dna sequencing, high-throughput” OR “illumina sequencing” OR “ion proton sequencing” OR “ion torrent sequencing” OR “massively parallel sequencing” OR “massively-parallel sequencing” OR “next generation sequencing” OR “nucleotide sequencing, high-throughput” OR “pyrosequencing” OR “sequenciamento de nucleotídeos em larga escala” OR “secuenciación de nucleótidos de alto rendimiento” OR “pirosecuenciación” OR “secuenciación ion proton” OR “secuenciación de alto rendimiento de nucleótidos” OR “secuenciación de illumina” OR “secuenciación de ion torrent” OR “secuenciación de nucleótidos a gran escala” OR “secuenciación de nucleótidos de alta productividad” OR “secuenciación de nueva generación” OR “piro-sequenciamento” OR pirossequenciamento OR “sequenciamento de nova geração” OR “sequenciamento de nucleotídeos de alta produtividade” OR “sequenciamento de nucleotídeos em grande escala”))) | 13  +  1 |
| Google Scholar Web Search | (“dental caries” OR “root caries” OR "Carious Dentin") AND ("sequence analysis, rna" OR RNA-sequencing OR "high-throughput nucleotide sequencing” OR "next generation sequencing" OR "High-Throughput RNA Sequencing" OR "High-Throughput DNA Sequencing" OR "High Throughput DNA Sequencing" OR "16S" OR "16S rRNA" OR "16S rDNA" OR “RNA, Ribosomal, 16S) | 7  +  0 |
| Proquest | noft("dental caries" OR "Dental Decay" OR "Carious Dentin" OR "Carious Dentins" OR "teeth carie" OR "teeth caries" OR "tooth caries" OR "teeth decay" OR "tooth decay" OR "dental decay" OR "Root Caries" OR "Cervical Caries") AND ("sequence analysis, rna" OR “RNA-sequencing” OR "high-throughput nucleotide sequencing" OR "high throughput nucleotide sequencing" OR "next generation sequencing" OR "Deep Sequencing" OR "High-Throughput RNA Sequencing" OR "High Throughput RNA Sequencing" OR "High-Throughput DNA Sequencing" OR "High Throughput DNA Sequencing" OR "16S" OR "16S rRNA" OR "16S rDNA" OR “RNA, Ribosomal, 16S") | 84 |
| Open Grey | microbiology AND "dental caries" | 8  +  0 |

**Appendix table 2.** Excluded articles and reasons for exclusion (n=64)

|  | **Author, year** | **Reason for exclusion** |
| --- | --- | --- |
| *After full-text reading* | | |
| 1 | (Abood and Richards 2018) | 5 |
| 2 | (Al-Ahmad, Auschill et al. 2016) | 8 |
| 3 | (Baik, Kwon et al. 2013) | 5 |
| 4 | (Boehlke, Rupf et al. 2020) | 7 |
| 5 | (Bruno, Heidrich et al. 2019) | 3 |
| 6 | (Carda-Diéguez, Bravo-González et al. 2020) | 7 |
| 7 | (Cephas, Kim et al. 2011) | 7 |
| 8 | (Chen, Zhang et al. 2019) | 7 |
| 9 | (Eriksson, Holgerson et al. 2018) | 5 |
| 10 | (Erin, Clifford et al. 2012) | 6 |
| 11 | (Esberg, Haworth et al. 2020) | 7 |
| 12 | (Funahashi, Shiba et al. 2019) | 3 |
| 13 | (Ge, Rodriguez et al. 2013) | 7 |
| 14 | (Gussy, Mnatzaganian et al. 2020) | 8 |
| 15 | (Harris-Ricardo, Fang et al. 2019) | 7 |
| 16 | (Hu, Huang et al. 2019) | 7 |
| 17 | (Johansson, Esberg et al. 2018) | 7 |
| 18 | (Johansson, Witkowska et al. 2016) | 1 |
| 19 | (Kazemtabrizi, Haddadi et al. 2020) | 8 |
| 20 | (Kianoush, Adler et al.) | 1 |
| 21 | (Milgrom, Horst et al. 2018) | 1 |
| 22 | (Nascimento, Alvarez et al. 2019) | 6 |
| 23 | (Nascimento, Alvarez et al. 2019) | 6 |
| 24 | (Ostrovska and Gerasimchuk 2019) | 4 |
| 25 | (Pozhitkov, Leroux et al. 2015) | 1 |
| 26 | (Prosdocimi, Kistler et al. 2017) | 6 |
| 27 | (Scott, Erik et al. 2013) | 6 |
| 28 | (Stsepetova, Truu et al. 2019) | 6 |
| 29 | (Sun, Xie et al. 2020) | 1 |
| 30 | (Takeshita, Kageyama et al. 2016) | 7 |
| 31 | (Thangam, Sathya Narayanan et al. 2017) | 3 |
| 32 | (Tu, He et al. 2014) | 8 |
| 33 | (Volgenant, Zaura et al. 2017) | 7 |
| 34 | (Yasunaga, Takeshita et al. 2017) | 7 |
| 35 | (Yue, Yin et al. 2018) | 3 |
| 36 | (Zheng, He et al. 2017) | 1 |
| 37 | (Lin, Bingcai et al. 2015) | 1 |
| 38 | (Chen, Shi et al. 2017) | 1 |
| 39 | (Du, Li et al. 2017) | 1 |
| 40 | (Gomar-Vercher, Cabrera-Rubio et al. 2014) | 1 |
| 41 | (He, Tu et al. 2018) | 1 |
| 42 | (Jiang, Gao et al. 2016) | 1 |
| 43 | (Jiang, Zhang et al. 2013) | 1 |
| 44 | (Jiang, Ling et al.) | 1 |
| 45 | (Kim, Han et al. 2018) | 1 |
| 46 | (Lee, Lee et al. 2016) | 1 |
| 47 | (Li, Tao et al.) | 1 |
| 48 | (Li, Zou et al. 2016) | 1 |
| 49 | (Ling, Kong et al. 2010) | 1 |
| 50 | (Marcelo, Ahmed et al. 2020) | 1 |
| 51 | (Mashima, Theodorea et al. 2019) | 1 |
| 52 | (Morou-Bermudez, Rodriguez et al. 2015) | 1 |
| 53 | (Nagai, Homma et al. 2020) | 1 |
| 54 | (Peterson, Meissner et al. 2014) | 1 |
| 55 | (Schoilew, Ueffing et al. 2019) | 1 |
| 56 | (Tao, Li et al. 2018) | 1 |
| 57 | (Uchida-Fukuhara, Ekuni et al. 2020) | 1 |
| 58 | (Wang, Wang et al. 2019) | 1 |
| 59 | (Yang, Zeng et al. 2012) | 1 |
| 60 | (Zhou, Jiang et al. 2016) | 1 |
| 61 | (Zhu, Yuan et al. 2018) | 1 |
| 62 | (Johansson, Witkowska et al. 2016) | 1 |
| 63 | (Pozhitkov, Leroux et al. 2015) | 1 |
| ***After methodological quality assessment*** | | |
| 64 | (Obata, Takeshita et al. 2014) | 10 |

1= Numeric data on either prevalence or abundance of *Veillonella* species not specifically described (author did not answer the protocol of data request, or denied sharing the data);

2 = Animals, *in situ* or *in vitro* studies;

3= Studies including systemic diseases or syndromes that can change the microbiota (Sjogren, severe hypossalivation, head and neck cancer, HIV, rheumatoid arthritis, asthma, alcoholism, etc);

4= Studies in languages not possible to be translated into an electronic translator;

5 = Articles with full text not available (Reviews, Book chapters, opinions, letters, conference abstracts, study protocols) or duplicate data;

6 = Studies where the protocol for sequencing includes either a cloning step or a previous treatment;

7 = No caries group;

8 = No NGS technique;

10 = “No” answers in highly critical domains of the Joanna Briggs Institute instrument.

**Excluded articles references:**

Abood, A. and V. P. Richards (2018). The Oral Microbiome of Site-Specific Dental Plaque in Health and Disease, Clemson University.

Al-Ahmad, A., T. M. Auschill, R. Dakhel, A. Wittmer, K. Pelz, C. Heumann, E. Hellwig and N. B. Arweiler (2016). "Prevalence of Candida albicans and Candida dubliniensis in caries-free and caries-active children in relation to the oral microbiota-a clinical study." Clin Oral Investig **20**(8): 1963-1971.

Baik, K. S., J. Kwon and J. H. Choi (2013). "Analysis of Oral Microbiota in Children’s Dental Caries." Toxicology and Environmental Health Sciences **5**: S59.

Boehlke, C., S. Rupf, M. Tenniswood, S. V. Chittur, C. Hannig and O. Zierau (2020). "Caries and periodontitis associated bacteria are more abundant in human saliva compared to other great apes." Arch Oral Biol **111**: 104648.

Bruno, J. S., V. Heidrich, F. H. Knebel, W. Miranda-Silva, A. A. Camargo and E. R. Fregnani (2019). "Characterization of oral and radiationrelated caries microbiota through metagenomic analysis: Preliminary results." Supportive Care in Cancer **27**(1): S148.

Carda-Diéguez, M., L. A. Bravo-González, I. M. Morata, A. Vicente and A. Mira (2020). "High-throughput DNA sequencing of microbiota at interproximal sites." J Oral Microbiol **12**(1): 1687397.

Cephas, K. D., J. Kim, R. A. Mathai, K. A. Barry, S. E. Dowd, B. S. Meline and K. S. Swanson (2011). "Comparative Analysis of Salivary Bacterial Microbiome Diversity in Edentulous Infants and Their Mothers or Primary Care Givers Using Pyrosequencing." Plos One **6**(8).

Chen, L., Q. Zhang, Y. Wang, K. Zhang and J. Zou (2019). "Comparing dental plaque microbiome diversity of extrinsic black stain in the primary dentition using Illumina MiSeq sequencing technique." BMC Oral Health **19**(1): 269.

Chen, T., Y. Shi, X. Wang, F. Meng, S. Yang, J. Yang and H. Xin (2017). "High-throughput sequencing analyses of oral microbial diversity in healthy people and patients with dental caries and periodontal disease." Molecular Medicine Reports **16**(1): 127-132.

Du, Q., M. Li, X. Zhou and K. Tian (2017). "A comprehensive profiling of supragingival bacterial composition in Chinese twin children and their mothers." Antonie Van Leeuwenhoek **110**(5): 615-627.

Eriksson, L., P. L. Holgerson, A. Esberg and I. Johansson (2018). "Microbial Complexes and Caries in 17-Year-Olds with and without Streptococcus mutans." Journal of Dental Research **97**(3): 275-282.

Erin, L. G., J. B. Clifford, R. K. Stacey, D. F. Noah, J. L. Eugene and L. G. Ann (2012). "Beyond Streptococcus mutans dental caries onset linked to multiple species by 16S rRNA community analysis." PLoS ONE, Vol 7, Iss 10, p e **47722**.

Esberg, A., S. Haworth, P. Hasslöf, P. Lif Holgerson and I. Johansson (2020). "Oral Microbiota Profile Associates with Sugar Intake and Taste Preference Genes." Nutrients **12**(3).

Funahashi, K., T. Shiba, T. Watanabe, K. Muramoto, Y. Takeuchi, T. Ogawa, Y. Izumi, T. Sekizaki, I. Nakagawa and K. Moriyama (2019). "Functional dysbiosis within dental plaque microbiota in cleft lip and palate patients." Prog Orthod **20**(1): 11.

Ge, X., R. Rodriguez, M. Trinh, J. Gunsolley and P. Xu (2013). "Oral microbiome of deep and shallow dental pockets in chronic periodontitis." PLoS One **8**(6): e65520.

Gomar-Vercher, S., R. Cabrera-Rubio, A. Mira, J. M. Montiel-Company and J. M. Almerich-Silla (2014). "Relationship of children's salivary microbiota with their caries status: a pyrosequencing study." Clin Oral Investig **18**(9): 2087-2094.

Gussy, M., G. Mnatzaganian, S. Dashper, L. Carpenter, H. Calache, H. Mitchell, E. Reynolds, L. Gibbs, S. Hegde, G. Adams, S. Johnson, E. Amezdroz and B. Christian (2020). "Identifying predictors of early childhood caries among Australian children using sequential modelling: Findings from the VicGen birth cohort study." J Dent **93**: 103276.

Harris-Ricardo, J., L. Fang, A. Herrera-Herrera, N. Fortich-Mesa, D. Olier-Castillo, D. Cavanzo-Rojas and R. González-Quintero (2019). "Bacterial profile of the supragingival dental biofilm in children with deciduous and early mixed dentition using next generation sequencing (HOMINGS) technique." Enferm Infecc Microbiol Clin **37**(7): 448-453.

He, J., Q. Tu, Y. Ge, Y. Qin, B. Cui, X. Hu, Y. Wang, Y. Deng, K. Wang, J. D. Van Nostrand, J. Li, J. Zhou, Y. Li and X. Zhou (2018). "Taxonomic and Functional Analyses of the Supragingival Microbiome from Caries-Affected and Caries-Free Hosts." Microb Ecol **75**(2): 543-554.

Hu, X., Z. Huang, Y. Zhang, Y. Hong and Y. Zheng (2019). "Effects of a probiotic drink containing Lactobacillus casei strain Shirota on dental plaque microbiota." J Int Med Res **47**(7): 3190-3202.

Jiang, S., X. Gao, L. Jin and E. C. Lo (2016). "Salivary Microbiome Diversity in Caries-Free and Caries-Affected Children." Int J Mol Sci **17**(12).

Jiang, W., Z. Ling, X. Lin, Y. Chen, J. Zhang, J. Yu, C. Xiang and H. Chen "Pyrosequencing Analysis of Oral Microbiota Shifting in Various Caries States in Childhood." Microbial ecology.

Jiang, W., J. Zhang and H. Chen (2013). "Pyrosequencing analysis of oral microbiota in children with severe early childhood dental caries." Curr Microbiol **67**(5): 537-542.

Johansson, I., A. Esberg, L. Eriksson, S. Haworth and P. Lif Holgerson (2018). "Self-reported bovine milk intake is associated with oral microbiota composition." PLoS One **13**(3): e0193504.

Johansson, I., E. Witkowska, B. Kaveh, P. Lif Holgerson and A. C. Tanner (2016). "The Microbiome in Populations with a Low and High Prevalence of Caries." J Dent Res **95**(1): 80-86.

Kazemtabrizi, A., A. Haddadi, M. Shavandi and N. Harzandi (2020). "Metagenomic investigation of bacteria associated with dental lesions: a cross-sectional study." Med Oral Patol Oral Cir Bucal **25**(2): e240-e251.

Kianoush, N., C. J. Adler, K.-A. T. Nguyen, G. V. Browne, M. Simonian and N. Hunter "Bacterial profile of dentine caries and the impact of pH on bacterial population diversity."

Kim, B. S., D. H. Han, H. Lee and B. Oh (2018). "Association of Salivary Microbiota with Dental Caries Incidence with Dentine Involvement after 4 Years." J Microbiol Biotechnol **28**(3): 454-464.

Lee, H. S., J. H. Lee, S. O. Kim, J. S. Song, B. I. Kim and Y. J. Kim (2016). "Comparison of the oral microbiome of siblings using next-generation sequencing: a pilot study." Oral Dis **22**(6): 549-556.

Li, F., D. Tao, X. Feng, M. C. M. Wong and H. Lu "Establishment and Development of Oral Microflora in 12-24 Month-Old Toddlers Monitored by High-Throughput Sequencing."

Li, Y., C. G. Zou, Y. Fu, Q. Zhou, B. Liu, Z. Zhang and J. Liu (2016). "Oral microbial community typing of caries and pigment in primary dentition." BMC Genomics **17**: 558.

Lin, C., Q. Bingcai, D. Minquan, Z. Huanzi, X. Qingan, L. Yuhong, Z. Ping and F. Mingwen (2015). "Extensive description and comparison of human supra-gingival microbiome in root caries and health." PLoS ONE, Vol 10, Iss 2, p e **117064**.

Ling, Z. X., J. M. Kong, P. Jia, C. C. Wei, Y. Z. Wang, Z. W. Pan, W. J. Huang, L. J. Li, H. Chen and C. Xiang (2010). "Analysis of Oral Microbiota in Children with Dental Caries by PCR-DGGE and Barcoded Pyrosequencing." Microbial Ecology **60**(3): 677-690.

Marcelo, F., M. Ahmed, M. H. Derek, G. T. Manolito, Z. Yun, L. Pamela, S. Richard, B. Michelle, K. Claire, H. Toby, M. C. Jeffrey and E. N. Karen (2020). "Longitudinal Study of Oral Microbiome Variation in Twins." Scientific Reports, Vol 10, Iss 1, Pp 1- **10**.

Mashima, I., C. F. Theodorea, B. Thaweboon, S. Thaweboon, T. Vichayanrat, F. A. Scannapieco and F. Nakazawa (2019). "Characterization of the salivary microbiome in healthy Thai children." Asian Pacific Journal of Tropical Medicine **12**(4): 163-169.

Milgrom, P., J. A. Horst, S. Ludwig, M. Rothen, B. W. Chaffee, S. Lyalina, K. S. Pollard, J. L. DeRisi and L. Mancl (2018). "Topical silver diamine fluoride for dental caries arrest in preschool children: A randomized controlled trial and microbiological analysis of caries associated microbes and resistance gene expression." J Dent **68**: 72-78.

Morou-Bermudez, E., S. Rodriguez, A. S. Bello and M. G. Dominguez-Bello (2015). "Urease and Dental Plaque Microbial Profiles in Children." PLoS One **10**(9): e0139315.

Nagai, N., H. Homma, A. Sakurai, N. Takahashi and S. Shintani (2020). "Microbiomes of colored dental biofilms in children with or without severe caries experience." Clin Exp Dent Res **6**(6): 659-668.

Nascimento, M. M., A. J. Alvarez, X. Huang, C. Browngardt, R. Jenkins, M. C. Sinhoreti, A. P. D. Ribeiro, D. A. Dilbone, V. P. Richards, T. J. Garrett and R. A. Burne (2019). "Metabolic Profile of Supragingival Plaque Exposed to Arginine and Fluoride." J Dent Res **98**(11): 1245-1252.

Nascimento, M. M., A. J. Alvarez, X. Huang, S. Hanway, S. Perry, A. Luce, V. P. Richards and R. A. Burne (2019). "Arginine Metabolism in Supragingival Oral Biofilms as a Potential Predictor of Caries Risk." JDR Clin Trans Res **4**(3): 262-270.

Obata, J., T. Takeshita, Y. Shibata, W. Yamanaka, M. Unemori, A. Akamine and Y. Yamashita (2014). "Identification of the Microbiota in Carious Dentin Lesions Using 165 rRNA Gene Sequencing." Plos One **9**(8).

Ostrovska, S. S. and P. G. Gerasimchuk (2019). "MEDICAL AND GENETIC STUDIES OF ORAL MICROBIOMA STATUS IN CHILDREN." ______ _______ ________ _ ________, Vol 1, Iss 4, Pp 43-- **7**.

Peterson, S. N., T. Meissner, A. I. Su, E. Snesrud, A. C. Ong, N. J. Schork and W. A. Bretz (2014). "Functional expression of dental plaque microbiota." Front Cell Infect Microbiol **4**: 108.

Pozhitkov, A. E., B. G. Leroux, T. W. Randolph, T. Beikler, T. F. Flemmig and P. A. Noble (2015). "Towards microbiome transplant as a therapy for periodontitis: an exploratory study of periodontitis microbial signature contrasted by oral health, caries and edentulism." BMC Oral Health **15**: 125.

Prosdocimi, E. M., J. O. Kistler, R. Moazzez, C. Thabuis, C. Perreau and W. G. Wade (2017). "Effect of maltitol-containing chewing gum use on the composition of dental plaque microbiota in subjects with active dental caries." J Oral Microbiol **9**(1): 1374152.

Schoilew, K., H. Ueffing, A. Dalpke, B. Wolff, C. Frese, D. Wolff and S. Boutin (2019). "Bacterial biofilm composition in healthy subjects with and without caries experience." J Oral Microbiol **11**(1): 1633194.

Scott, N. P., S. Erik, L. Jia, C. O. Ana, K. Mogens, J. S. Nicholas and B. Walter (2013). "The dental plaque microbiome in health and disease." PLoS ONE, Vol 8, Iss 3, p e **58487**.

Stsepetova, J., J. Truu, R. Runnel, R. Nommela, M. Saag, J. Olak, H. Nolvak, J. K. Preem, K. Oopkaup, K. Krjutskov, E. Honkala, S. Honkala, K. Makinen, P. L. Makinen, T. Vahlberg, J. Vermeiren, D. Bosscher, P. de Cock and R. Mandar (2019). "Impact of polyols on Oral microbiome of Estonian schoolchildren." Bmc Oral Health **19**.

Sun, C., Y. Xie, X. Hu, J. Fu, J. Zhou and L. Wu (2020). "Relationship between Clinical Symptoms and the Microbiota in Advanced Caries." J Endod **46**(6): 763-770.

Takeshita, T., S. Kageyama, M. Furuta, H. Tsuboi, K. Takeuchi, Y. Shibata, Y. Shimazaki, S. Akifusa, T. Ninomiya, Y. Kiyohara and Y. Yamashita (2016). "Bacterial diversity in saliva and oral health-related conditions: the Hisayama Study." Sci Rep **6**: 22164.

Tao, D., F. Li, X. Feng, M. C. M. Wong and H. Lu (2018). "Plaque biofilm microbial diversity in infants aged 12 months and their mothers with or without dental caries: a pilot study." BMC Oral Health **18**(1): 228.

Thangam, M., G. Sathya Narayanan, B. Rayvathy and J. Stalin Roy (2017). "Characterisation of the human oral microbiome in patients with coronary artery disease using next-generation sequencing of 16SrRNA amplicons." Indian Journal of Medical Microbiology, Vol 35, Iss 1, Pp 101- **104**.

Tu, Q., Z. He, Y. Li, Y. Chen, Y. Deng, L. Lin, C. L. Hemme, T. Yuan, J. D. Van Nostrand, L. Wu, X. Zhou, W. Shi, L. Li, J. Xu and J. Zhou (2014). "Development of HuMiChip for functional profiling of human microbiomes." PLoS One **9**(3): e90546.

Uchida-Fukuhara, Y., D. Ekuni, M. M. Islam, K. Kataoka, A. Taniguchi-Tabata, D. Fukuhara, N. Toyama, T. Kobayashi, K. Fujimori, N. Sawada, Y. Iwasaki and M. Morita (2020). "Caries Increment and Salivary Microbiome during University Life: A Prospective Cohort Study." Int J Environ Res Public Health **17**(10).

Volgenant, C. M., E. Zaura, B. W. Brandt, M. J. Buijs, M. Tellez, G. Malik, A. I. Ismail, J. M. Ten Cate and M. H. van der Veen (2017). "Red fluorescence of dental plaque in children -A cross-sectional study." J Dent **58**: 40-47.

Wang, Y., S. Wang, C. Wu, X. Chen, Z. Duan, Q. Xu, W. Jiang, L. Xu, T. Wang, L. Su, Y. Chen, J. Zhang, Y. Huang, S. Tong, C. Zhou, S. Deng and N. Qin (2019). "Oral Microbiome Alterations Associated with Early Childhood Caries Highlight the Importance of Carbohydrate Metabolic Activities." mSystems **4**(6).

Yang, F., X. Zeng, K. Ning, K. L. Liu, C. C. Lo, W. Wang, J. Chen, D. Wang, R. Huang, X. Chang, P. S. Chain, G. Xie, J. Ling and J. Xu (2012). "Saliva microbiomes distinguish caries-active from healthy human populations." ISME J **6**(1): 1-10.

Yasunaga, H., T. Takeshita, Y. Shibata, M. Furuta, Y. Shimazaki, S. Akifusa, T. Ninomiya, Y. Kiyohara, I. Takahashi and Y. Yamashita (2017). "Exploration of bacterial species associated with the salivary microbiome of individuals with a low susceptibility to dental caries." Clin Oral Investig **21**(8): 2399-2406.

Yue, Q., F. T. Yin, Q. Zhang, C. Yuan, M. Y. Ye, X. L. Wang, J. J. Li and Y. H. Gan (2018). "Carious status and supragingival plaque microbiota in hemodialysis patients." PLoS One **13**(10): e0204674.

Zheng, X., J. He, L. Wang, S. Zhou, X. Peng, S. Huang, L. Zheng, L. Cheng, Y. Hao, J. Li, J. Xu, X. Xu and X. Zhou (2017). "Ecological Effect of Arginine on Oral Microbiota." Sci Rep **7**(1): 7206.

Zhou, J., N. Jiang, S. Wang, X. Hu, K. Jiao, X. He, Z. Li and J. Wang (2016). "Exploration of human salivary microbiomesâ€"insights into the novel characteristics of microbial community structure in caries and caries-free subjects." PLoS ONE **11**(1).

Zhu, C., C. Yuan, S. Ao, X. Shi, F. Chen, X. Sun and S. Zheng (2018). "The Predictive Potentiality of Salivary Microbiome for the Recurrence of Early Childhood Caries." Front Cell Infect Microbiol **8**: 423.

**Appendix table 3.** Assessment of methodological quality of individual studies using the JBI Critical Appraisal Checklist for Analytical Cross-Sectional Studies (Moola et al. 2020).

| Author, year | 1. Were the criteria for inclusion in the sample clearly defined? | 2. Were the study subjects and the setting described in detail? | **3. Was the exposure measured in a valid and reliable way?** | **4. Were objective, standard criteria used for measurement of the condition?** | 5. Were confounding factors identified? | 6. Were strategies to deal with confounding factors stated? | 7. Were the outcomes measured in a valid and reliable way? | 8. Was appropriate statistical analysis used? | **Overall appraisal:**  LOW, MODERATE, OR HIGH QUALITY |
| --- | --- | --- | --- | --- | --- | --- | --- | --- | --- |
| Agnello, 2017 | U | Y | **U** | **U** | Y | Y | Y | Y | **LOW** |
| Al-Hebshi, 2019 | Y | N | **Y** | **U** | Y | U | Y | Y | **LOW** |
| Belstram, 2017 | Y | Y | **Y** | **Y** | U | U | Y | U | **HIGH** |
| Corralo, 2018 | Y | N | **Y** | **Y** | Y | U | Y | Y | **HIGH** |
| Dame-Teixeira, 2020 | U | N | **Y** | **Y** | U | U | Y | Y | **LOW** |
| Dashper, 2019 | U | N | **Y** | **Y** | U | U | Y | Y | **LOW** |
| Do, 2015 | U | N | **U** | **U** | U | U | Y | Y | **LOW** |
| Eriksson, 2017 | Y | Y | **Y** | **U** | Y | U | Y | Y | **LOW** |
| Jagathrakshakan, 2015 | U | N | **U** | **U** | U | U | Y | Y | **LOW** |
| Jiang, 2019 | Y | N | **Y** | **Y** | Y | U | Y | Y | **MODERATE** |
| Meng, 2015 | Y | N | **Y** | **Y** | Y | U | Y | Y | **MODERATE** |
| Ribeiro, 2017 | Y | N | **Y** | **Y** | U | U | Y | Y | **LOW** |
| Roças, 2016 | U | N | **Y** | **Y** | U | U | Y | Y | **LOW** |
| Xiao, 2016 | Y | Y | **Y** | **Y** | Y | U | Y | Y | **HIGH** |
| Xiao, 2018 | U | Y | **Y** | **Y** | Y | Y | Y | Y | **HIGH** |
| Xu, Chen, 2018 | Y | Y | **Y** | **Y** | Y | U | Y | Y | **MODERATE** |
| Xu, Jia, 2018 | Y | N | **U** | **U** | U | U | Y | Y | **LOW** |
| Nomura, 2020 | U | N | **Y** | **U** | U | U | Y | Y | **LOW** |
| Yun, 2019 | Y | Y | **Y** | **U** | Y | U | Y | Y | **LOW** |
| Ortiz, 2019 | Y | Y | **Y** | **U** | U | U | Y | U | **LOW** |
| Richards, 2017 | Y | Y | **Y** | **Y** | U | U | Y | U | **MODERATE** |
| Zheng, 2018 | Y | Y | **Y** | **U** | U | U | Y | U | **LOW** |
| Simon-Soro, 2014 | U | N | **U** | **U** | Y | Y | Y | Y | **LOW** |
| Simon-Soro, 2013 | U | N | **U** | **U** | Y | Y | Y | Y | **LOW** |
| Hurley, 2019 | Y | N | **U** | **U** | Y | Y | Y | Y | **LOW** |
| Alcaraz, 2012 | U | N | **U** | **U** | U | U | U | Y | **LOW** |
| Belda-Ferre, 2012 | N | N | **U** | **U** | N | N | Y | Y | **LOW** |
| Wolff, 2019 | N | N | **U** | **U** | Y | Y | Y | Y | **LOW** |
| de Jesus, 2020 | Y | Y | **Y** | **Y** | U | U | Y | Y | **MODERATE** |
| Foxman, 2016 | U | Y | **U** | **U** | U | U | Y | U | **LOW** |
| Xu and Hao, 2014 | Y | Y | **Y** | **Y** | U | U | Y | U | **MODERATE** |
| Tian, 2015 | U | Y | **U** | **U** | U | U | Y | Y | **LOW** |
| Xu and Tian, 2018 | Y | Y | **Y** | **Y** | Y | U | Y | Y | **HIGH** |
| Chen, 2020 | Y | N | **Y** | **U** | Y | Y | Y | Y | **MODERATE** |
| Grier, 2020 | N | N | **U** | **Y** | U | U | Y | Y | **LOW** |
| Qudeimat, 2021 | Y | Y | **Y** | **U** | Y | Y | Y | Y | **HIGH** |
| Schulze-Schweifing, 2012 (page 190) | Y | N | **U** | **U** | U | U | Y | Y | **LOW** |
| Mitwalli, 2019 | Y | Y | **Y** | **Y** | Y | Y | Y | U | **HIGH** |

1. Were the criteria for inclusion in the sample clearly defined? **CRITICAL DOMAIN**

2. Were the study subjects and the setting described in detail? **CRITICAL DOMAIN**

3. Was the exposure measured in a valid and reliable way? **VERY CRITICAL DOMAIN**

4. Were objective, standard criteria used for measurement of the condition?**VERY CRITICAL DOMAIN**

5. Were confounding factors identified? **NON-CRITICAL DOMAIN**

6. Were strategies to deal with confounding factors stated? **NON-CRITICAL DOMAIN**

7. Were the outcomes measured in a valid and reliable way? **NON-CRITICAL DOMAIN**

8. Was appropriate statistical analysis used? **NON-CRITICAL DOMAIN**

**Appendix table 4.** GRADE summary of findings table for *Veillonella* spp. abundance in ECC individuals compared to caries-free individuals.

| **Outcomes** | **№ of participants  (studies) Follow up** | **Certainty of the evidence (GRADE)** | **Abundance mean difference for *Veillonella* spp. average** |
| --- | --- | --- | --- |
|  |  |  |  |
| *Veillonella* spp. average abundance in ECC | 319 (6 observational studies) | ⨁◯◯◯ VERY LOW ^a^ | MD **2.22 higher** (0.54 higher to 3.9 higher) |
| * CI: Confidence interval; MD: Mean difference  a. Risk of bias dowgraded in one point since the removal of two low-quality studies during sensitivity analysis altered the MA result (MD 2.07 CI 95% -2.44 to 6.58, P>0.05) | | | |
